# PEPR1 Mediates SsNLP1-Triggered Immunity Against *Sclerotinia sclerotiorum*

**DOI:** 10.3390/ijms27125271

**Published:** 2026-06-10

**Authors:** Imtiaz Ahmad Sajid, Muhammad Kamran, Zeeshan Ghulam Nabi Gishkori, Xin-Zhong Cai

**Affiliations:** 1 Zhejiang Key Laboratory of Biology and Ecological Regulation of Crop Pathogens and Insects, Institute of Biotechnology, College of Agriculture and Biotechnology, Zhejiang University, 866 Yu Hang Tang Road, Hangzhou 310058, China; 2 Institute of Crop Science, College of Agriculture and Biotechnology, Zhejiang University, Hangzhou 310058, China; 3 Hainan Institute, Zhejiang University, Sanya 572025, China

**Keywords:** *Sclerotinia sclerotiorum*, SsNLP1, PEPR1, pattern-triggered immunity, resistance

## Abstract

Necrosis- and ethylene-inducing peptide 1 (Nep1)-like proteins (NLPs) are conserved microbial proteins that contain immunogenic patterns capable of activating plant pattern-triggered immunity (PTI). NLP patterns from *Sclerotinia sclerotiorum* (SsNLPs), a destructive necrotrophic fungal pathogen with a broad host range, have been identified, and their roles in PTI have been revealed. Nevertheless, the molecular mechanisms by which SsNLPs stimulate plant immunity remain largely unknown. In this study, we phylogenetically characterized SsNLPs and demonstrated the involvement of the phytocytokine receptor-like kinases PEPRs in SsNLP1-triggered immunity. SsNLPs contained the NPP1 domain and GHRHDWE motif and were phylogenetically closely associated with *Botrytis cinerea* NLPs. SsNLP1 treatment strongly induced the expression of *PEPR* genes. Further genetic analyses using *Arabidopsis* wild-type, *pepr1 pepr2* double mutant, and *PEPR1* overexpression lines showed that SsNLP1 elicited diverse immune responses, including reactive oxygen species (ROS) accumulation and defense gene activation, and induced plant resistance to *S. sclerotiorum*. Notably, the induced plant resistance and immune responses were strengthened in *PEPR1* overexpression lines and significantly reduced in the *pepr1 pepr2* mutant, indicating a positive role of PEPR signaling in SsNLP1-triggered immunity. Overall, our results revealed that phytocytokine PEPR1 signaling amplifies PAMP SsNLP1-triggered immunity, thereby enhancing resistance against *S. sclerotiorum*. Our findings provide an example of the coordination between PAMP- and phytocytokine-triggered immunity for robust resistance to a necrotrophic pathogen.

## 1. Introduction

Plants have evolved a sophisticated innate immune system to counteract pathogen invasion. Traditionally, plant immunity has been described as comprising pattern-triggered immunity (PTI) and effector-triggered immunity (ETI). PTI is activated upon recognition of conserved pathogen- or microbe-associated molecular patterns (PAMPs/MAMPs) by plasma membrane-localized pattern recognition receptors (PRRs), whereas ETI is induced following detection of pathogen-derived effectors by intracellular nucleotide-binding leucine-rich repeat (NLR) receptors [1,2]. Recent studies have demonstrated that PTI and ETI are highly interconnected and function through extensive crosstalk and mutual potentiation, with activation of one pathway enhancing the strength and effectiveness of the other to promote robust disease resistance [3,4,5]. PRRs include receptor-like kinases (RLKs) and receptor-like proteins (RLPs), the latter lacking an intracellular kinase domain but functioning through association with co-receptors to initiate downstream signaling [6,7,8]. Well-characterized examples include FLS2 and EFR, which perceive bacterial flagellin (flg22) and elongation factor Tu (elf18), respectively, in association with the co-receptor BAK1 [9,10]. Similarly, LYK5 recognizes fungal chitin together with CERK1 [11], while RLP23 detects conserved nlp20/nlp24 peptide motifs derived from necrosis- and ethylene-inducing peptide 1 (Nep1)-like proteins (NLPs), which are broadly present in bacterial, fungal, and oomycete pathogens [12,13].

These NLP-derived peptides have been functionally characterized in several pathogens, including the oomycete *Hyaloperonospora arabidopsidis* [14,15] and the fungal pathogen *Botrytis cinerea* [16]. A conserved heptapeptide motif, GHRHDWE, is essential for NLP function and is required for triggering immune responses in plants [17]. Recognition of nlp20 by RLP23 involves formation of a receptor complex with SOBIR1 and the co-receptor BAK1 [18]. Additionally, *Sclerotinia sclerotiorum* also secretes two NLP-type proteins, SsNEP1 and SsNEP2, which have been functionally characterized as necrosis- and ethylene-inducing proteins [19]. Recently, we identified two nlp24-type PAMPs, SsNLP1 and SsNLP2, from SsNEP1 and SsNEP2, and demonstrated that they trigger RLP23-dependent immunity in *Arabidopsis* and oilseed rape. We further revealed that SsNLPs activate differential phosphorylation of the transcription factor WRKY8 by CPK4/CPK11 and PBL19 to simultaneously amplify *RLP23* and *CERK1* expression for broad-spectrum immunity [20]. Nevertheless, the molecular mechanisms underlying SsNLP-triggered immunity require further exploration.

In addition to recognizing pathogen-derived molecular patterns, plants also activate immune responses through endogenous danger signals known as damage-associated molecular patterns (DAMPs) and phytocytokines, which are produced upon pathogen infection or cellular damage [21,22]. Among the best-characterized phytocytokines are plant elicitor peptides (PEPs), which are perceived by two specific PRRs, PEP receptor 1 (PEPR1) and PEPR2 [23]. They are closely related leucine-rich repeat receptor-like kinase (LRR-RLK) PRRs for endogenous *Arabidopsis* PEPs and play important roles in plant immunity [24,25]. Phytocytokine pathways are extensively interconnected. Reciprocal transcriptional amplification occurs between PIP1 and PEP1 precursors [26]. Furthermore, RALF22 perception strongly induces *PROPEP3* expression, thereby potentiating PEP3-mediated immunity [27]. PEPR1- and PEPR2-mediated signaling is required for plant resistance against various pathogens such as *B. cinerea* [28], *Pseudomonas syringae* pv. *tomato* (*Pst*) [29], *Pythium irregulare* [25], as well as insect herbivory [30]. However, whether it is involved in SsNLPs-triggered immunity and resistance to *S. sclerotiorum* remains unclear.

It is well known that the PRR RLP23 directly recognizes conserved NLP-derived peptides such as nlp20; however, the signaling downstream of this perception remains largely unknown. Perception of fungal NLPs by RLP23 may be associated with host cell damage and the consequent release of endogenous phytocytokines. This raises the possibility that NLP- and phytocytokine-triggered signaling may coordinate to promote robust plant resistance. Whether SsNLP signaling coordinates with PEPR signaling for strong plant resistance to *S. sclerotiorum* is thus worthy of experimental verification.

In this study, we characterize NLPs from *S. sclerotiorum* and functionally evaluate the contribution of PEPR signaling to SsNLP1-triggered immunity and resistance to *S. sclerotiorum*. Our data reveal the coordinative role of phytocytokine signaling with PAMP signaling in enhanced plant resistance to *S. sclerotiorum*, thus providing insights into plant immunity and disease resistance.

## 2. Results

### 2.1. Phylogenetic and Structural Characterization of NLP Family Proteins Across Pathogen Species

NLPs are widely distributed across bacterial, fungal, and oomycete microbes and are defined by a conserved NPP1 domain (Pfam PF05630) and a hallmark GHRHDWE heptapeptide motif, which is essential for immune elicitation [12,17]. To characterize the NLP family across major plant pathogens, we performed BLASTp-based identification of NLP sequences from 15 pathogen species spanning fungi (Ascomycota) and oomycetes (Oomycota). All NLP paralogs were retained per species to capture the complete NLP repertoire of each pathogen. The number of NLP family members varied across species: three in *Fusarium oxysporum* (FoNLP1–3), *Verticillium dahliae* (VdNLP1–3), and *Aspergillus vitricola* (AvNLP1–3); two in *S. sclerotiorum* (SsNLP1–2), *B. cinerea* (BcNLP1–2), *Aspergillus destruens* (AdNLP1–2) and *Cladosporium cladosporioides* (CcNLP1–2); and one in *Aspergillus magnivesiculatus*, *A. proliferans*, *Penicillium chrysogenum*, *Colletotrichum gloeosporioides*, *Fulvia fulva*, *Cladobotryum mycophilum*, *Phytophthora parasitica*, *Pythium aphanidermatum*, and *Pyricularia oryzae.* In total, 26 NLP sequences were subjected to phylogenetic analysis.

**Figure 1:**
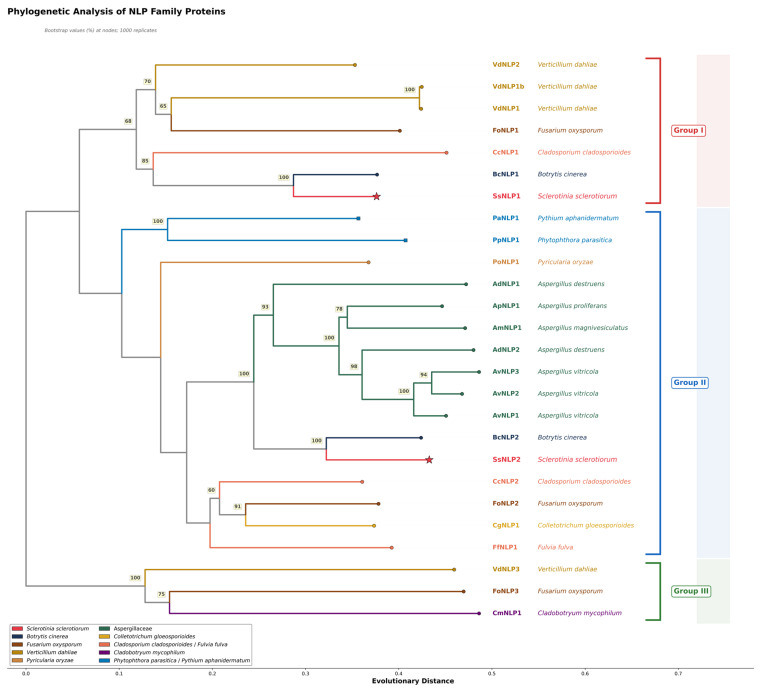
Phylogenetic analysis of NLP family proteins across pathogen species. Neighbor-joining tree of 26 NLP sequences from 15 fungal and oomycetes species. Bootstrap support values (1000 replicates) are shown at nodes (≥50%). Three phylogenetic groups are indicated by colored brackets: Group I (red), primary NLP paralogs of necrotrophic fungi; Group II (blue), oomycete NLPs, Aspergillaceae NLPs, second paralogs, and single NLPs; and Group III (green), third NLP paralogs and CmNLP1. Symbols at the ends of the tree clades indicate the NLP source pathogens: Stars indicate *S. sclerotiorum* NLPs; full circles indicate other fungi; and squares indicate oomycetes. Scale bar indicates evolutionary distance.

Neighbor-joining phylogenetic analysis revealed that the 26 NLPs were organized into three major groups (Figure 1, Table 1). Group I comprised NLPs from necrotrophic Leotiomycetes and Sordariomycetes pathogens, including SsNLP1 with BcNLP1 (100% bootstrap), FoNLP1 with VdNLP1, VdNLP1b, and VdNLP2 (66–100% bootstrap), and CcNLP1, forming a well-supported clade (84% bootstrap) that unites the primary NLP paralogs of these broad-host-range necrotrophic fungi. Group II contained phylogenetically diverse NLPs, including the oomycete NLPs from *Phytophthora parasitica* and *Pythium aphanidermatum*, and the divergent fungal NLPs from Aspergillaceae (AvNLP1-3, AdNLP1-2, AmNLP1, ApNLP1 and PcNLP1), together with the second NLP paralogs from Sclerotiniaceae (BcNLP2 and SsNLP2) and others (FoNLP2 and CcNLP2), as well as the single NLPs (CgNLP1 and FfNLP1). Notably, oomycete NLPs formed a distinct sub-clade separate from the second NLP paralogs from several fungal NLPs, implying that duplication of fungal NLPs predated the evolutionary divergence between these two kingdoms. Group III consisted of the third NLP paralogs from *Fusarium oxysporum* (FoNLP3) and *Verticillium dahliae* (VdNLP3), and the single NLP from *Cladobotryum mycophilum* (CmNLP1). Except for *Aspergillus* species, NLP paralogs of the same pathogen species existed in different groups, indicating that NLP gene duplication was followed by functional divergence within individual pathogen genomes.

**Figure 2:**
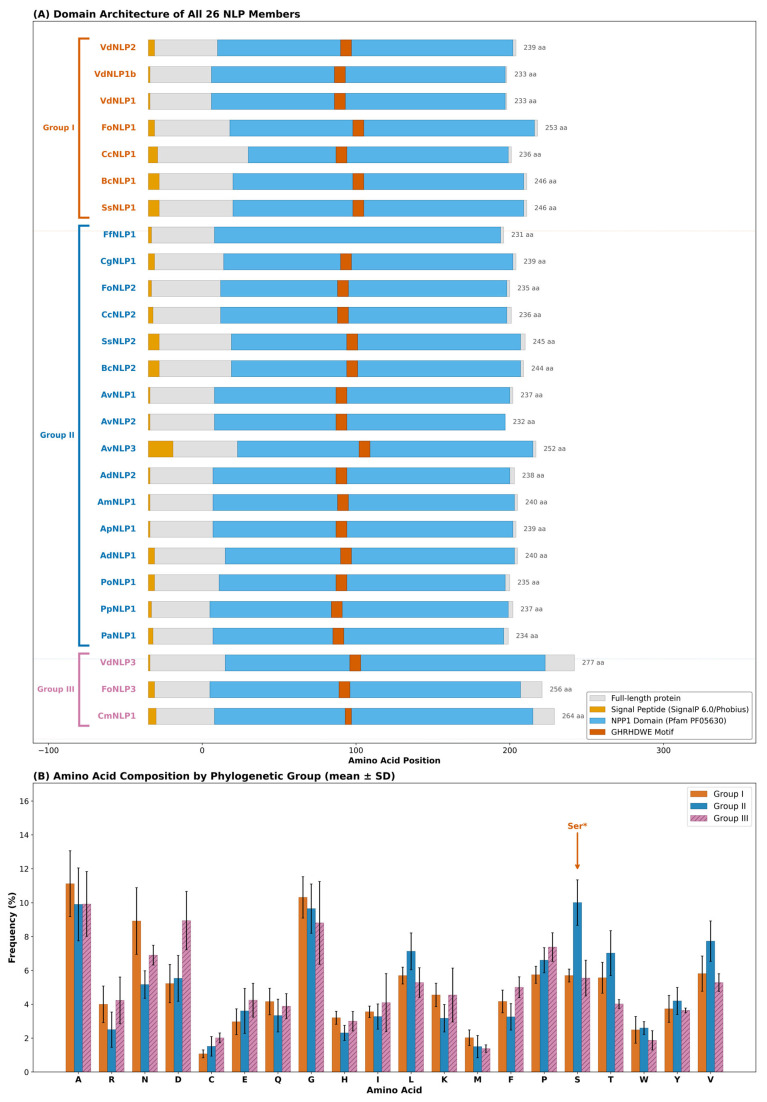
Domain architecture and amino acid composition of NLP family members. (**A**) Domain architecture of all 26 NLPs organized by phylogenetic group (brackets: orange, Group I; blue, Group II; reddish purple, Group III). Gray bars represent the full-length proteins, among which signal peptide (orange; SignalP 6.0/Phobius), NPP1 domain (sky blue; Pfam PF05630), and GHRHDWE motif (vermillion) are shown. All boundaries were verified by InterProScan. A colorblind-accessible palette (Okabe–Ito) was used. (**B**) Mean amino acid frequency (±SD) by phylogenetic group. Letters denote the names of amino acids. Group I (orange), Group II (blue), and Group III (reddish purple with hatching) were compared. Vermillion arrow highlights higher serine (indicated by *) in Group II (10.0 ± 1.3%) than in Group I (5.7 ± 0.4%) and in Group III (5.5 ± 0.9%).

InterProScan-based domain analysis of all 26 NLPs confirmed that each protein contains the diagnostic NPP1 domain (Pfam PF05630; Figure 2A). Signal peptide was predicted by SignalP 6.0 in 23 of the 26 NLPs, with three exceptions: SsNLP2, AvNLP3, and FfNLP1. However, they were predicted to contain signal peptides by Phobius, suggesting unconventional secretion signals in these proteins. Signal peptide lengths varied considerably, ranging from 14 aa (FoNLP2, ApNLP1, AvNLP1) to 35 aa (AvNLP3), with most NLPs having SP lengths of 17–21 aa. The NPP1 domain spanned the majority of NLPs. The GHRHDWE motif was present in 24 of 26 NLPs at positions ranging from 120 (PpNLP1) to 138 (AvNLP3). Notably, two NLPs, CmNLP1 (*Cladobotryum mycophilum*) and FfNLP1 (*F. fulva*), lacked the canonical GHRHDWE motif despite retaining the NPP1 domain fold. CmNLP1 contained a partial variant (GHRH at position 129). These results suggest that these proteins may have divergent immune-eliciting properties or have acquired alternative biological functions.

Amino acid composition analysis revealed both conserved features and notable differences among NLP groups (Figure 2B). All NLPs were enriched in glycine (8–10%), alanine (9–11%), and asparagine (5–8%), consistent with the hydrophilic surface properties characteristic of secreted effector proteins. A prominent difference was observed in serine content: Group II NLPs showed markedly higher serine frequency (10.0 ± 1.3%) compared with Group I (5.7 ± 0.4%) and Group III (5.5 ± 0.9%). This elevated serine content in Group II, which includes the second NLP paralogs, Aspergillaceae NLPs, and oomycete NLPs, may reflect distinct post-translational modification patterns, as serine residues serve as potential phosphorylation sites that could influence protein stability or host recognition properties. Group III NLPs also contained more threonine, valine, and leucine. Group I NLPs carried more asparagine, while Group III NLPs contained more aspartic acid. Additionally, cysteine content was highest in Group III (2.0 ± 0.2%) compared with Group I (1.1 ± 0.2%) and Group II (1.5 ± 0.6%), with Group III members (VdNLP3, FoNLP3) containing 4–6 cysteines, characteristic of Type II NLPs with additional disulfide bonds, whereas Group I NLPs typically contained 2–3 cysteines consistent with Type I NLP classification. SsNLP1 and SsNLP2 shared only 39.0% pairwise identity despite belonging to the same pathogen species, with SsNLP1 carrying a higher net positive charge (+27 versus +17 for SsNLP2), a difference that could influence their membrane interaction properties.

### 2.2. SsNLP1 Induces the Expression of PEPR1 and PEPR2 in Arabidopsis

**Figure 3:**
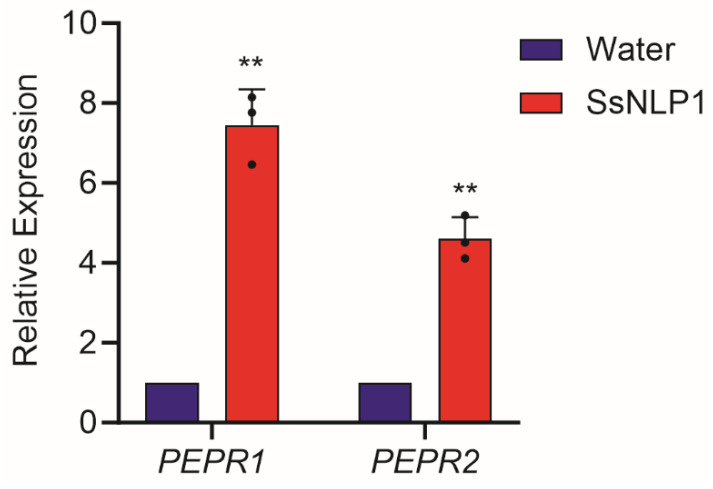
SsNLP1 induces expression of *PEPR1* and *PEPR2* in *Arabidopsis*. Leaves were treated with 1 μM SsNLP1 or water (mock control) for 1 h, and transcript levels were quantified by qRT-PCR. Data represent the mean ± SD (*n* = 3). Statistical significance was determined using Student’s *t*-test (** *p* < 0.01).

The nlp24-type peptides SsNLP1 and SsNLP2 were recently identified as active PAMPs that trigger RLP23-dependent immunity to *S. sclerotiorum* [20]. To investigate whether SsNLP1-induced immune signaling also engages endogenous danger–response pathways, we tested whether the peptide affects the expression of the phytocytokine receptors PEPR1 and PEPR2. To this end, leaves from Col-0 (WT) plants were infiltrated with 1 μM SsNLP1, with water treatment serving as the mock control (Figure 3). At 1 h post-treatment, transcript levels of *PEPR1* and *PEPR2* were quantified by quantitative real-time PCR (qRT-PCR).

Both *PEPR1* and *PEPR2* showed increased expression following SsNLP1 treatment compared with the control. *PEPR1* and *PEPR2* transcript levels increased by approximately 7.4-fold and 4.6-fold, respectively. The induction of *PEPR1* was greater than that of *PEPR2* under the same treatment conditions. These results indicate that SsNLP1 promotes the expression of PEPR receptors, with a relatively stronger effect on PEPR1. This finding provides a rationale for examining the role of PEPR1 in SsNLP1-triggered immunity and resistance, and it suggests that PEPR-mediated signaling may represent a pathway of immune signal amplification downstream of the well-established RLP23-dependent perception of PAMP NLPs [20].

### 2.3. PEPR1 Augments SsNLP1-Induced Resistance to S. sclerotiorum

**Figure 4:**
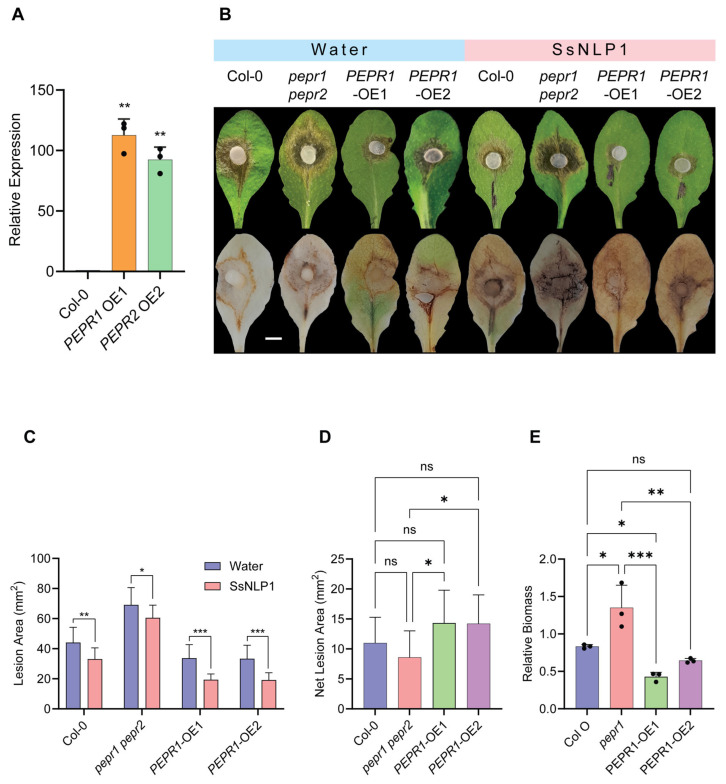
PEPR1 augments SsNLP1-induced resistance to *S. sclerotiorum*. (**A**) Validation of *PEPR1* overexpression lines in *Arabidopsis*. Expression analysis of *PEPR1* in *Arabidopsis* Col-0 and two independent overexpression lines (*PEPR1*-OE1 and *PEPR1*-OE2). The expression level of *PEPR1* in Col-0 was set to 1. Transcript abundance was quantified by qRT-PCR. Data represent the mean ± SD (*n* = 3). Statistical significance was determined using Student’s *t*-test. (**B**–**E**) Functional analysis of PEPR1 in SsNLP1-induced resistance against *S. sclerotiorum.* Disease progression and fungal biomass were analyzed in *Arabidopsis* Col-0, the *pepr1 pepr2* double mutant, and *PEPR1*-OE lines following SsNLP1 pre-treatment. Leaves were pre-treated via infiltration with either 1 μM SsNLP1 or water (mock) and inoculated with *S. sclerotiorum* mycelial plugs at 24 h post-treatment. Disease symptoms were recorded and analyzed at 24 h post-inoculation. (**B**) Representative images of infected leaves showing disease symptoms in different genotypes under water and SsNLP1 pre-treatment conditions. Scale bar = 5 mm. (**C**) Quantification of lesion area showing changes in disease severity following SsNLP1 pre-treatment compared with water treatment across genotypes. Statistical significance was determined using Student’s *t*-test (*n* = 9–12). (**D**) Net lesion reduction calculated as the difference between water- and SsNLP1-treated samples, illustrating the magnitude of SsNLP1-associated disease attenuation in each genotype. Statistical significance was determined using one-way ANOVA. (**E**) Relative fungal biomass in SsNLP1-infiltrated leaves relative to water-infiltrated leaves, based on qPCR detection of fungal DNA from infected leaf tissues (*n* = 3). Statistical significance was determined using one-way ANOVA. Data are expressed as the mean ± SD. Statistical significance is indicated as * *p* < 0.05, ** *p* < 0.01, *** *p* < 0.001, and ns, not significant.

To examine the role of PEPR1 in SsNLP1-mediated immunity, two independent *PEPR1* overexpression (OE) lines (35S::PEPR1-HA) were generated and validated. Transcript analysis confirmed strong upregulation of *PEPR1*, with *PEPR1*-OE1 showing approximately 112-fold and *PEPR1*-OE2 approximately 94-fold higher expression relative to Col-0 (Figure 4A). The *pepr1 pepr2* double mutant used in this study has been previously characterized and confirmed to lack both PEPR1 and PEPR2 function [31].

Leaves of *Arabidopsis* Col-0, the *pepr1 pepr2* double mutant, and two independent *PEPR1* OE lines were pre-treated via infiltration with either 1 μM SsNLP1 or water (mock) and maintained for 24 h prior to inoculation with *S. sclerotiorum* mycelial plugs. Disease development was assessed by imaging and lesion measurement at 24 h post-inoculation (Figure 4B–D). Visual assessment of infected leaves (Figure 4B, upper panel) showed differences in disease progression among genotypes and treatments. SsNLP1 pre-treatment generally reduced lesion development compared with water treatment, with a more pronounced effect in *PEPR1*-OE lines, whereas the *pepr1 pepr2* mutant exhibited increased susceptibility. Quantification of lesion area (Figure 4C) supported these observations, showing reduced disease severity following SsNLP1 pre-treatment across genotypes. The reduction was more evident in *PEPR1*-OE lines, while the *pepr1 pepr2* mutant showed comparatively increased lesion development and a weaker response to SsNLP1. To normalize treatment effects, net lesion reduction (water minus SsNLP1) was calculated (Figure 4D). This analysis indicated that SsNLP1-associated protection was highest in *PEPR1*-OE lines, intermediate in Col-0, and lowest in the *pepr1 pepr2* mutant, suggesting a contribution of PEPR1 to the magnitude of SsNLP1-induced resistance.

Fungal biomass was further quantified using qPCR-based detection of *S. sclerotiorum* DNA (Figure 4E). Consistent with lesion measurements, SsNLP1 pre-treatment reduced fungal biomass across all genotypes, with the strongest reduction observed in *PEPR1*-OE lines. In contrast, the *pepr1 pepr2* mutant accumulated higher fungal biomass, indicating enhanced susceptibility. Fungal biomass closely matched disease symptom development, supporting the reliability of the phenotypic observations.

Overall, these results indicate that PEPR1 signaling contributes to SsNLP1-induced plant resistance to *S. sclerotiorum*.

### 2.4. PEPR1 Contributes to SsNLP1-Triggered ROS Production and Defense Gene Expression

To investigate the contribution of PEPR1 to the early immune responses triggered by SsNLP1, ROS accumulation and defense-related gene expression after SsNLP1 treatment were analyzed in *Arabidopsis* Col-0, the *pepr1 pepr2* double mutant, and *PEPR1*-OE lines.

**Figure 5:**
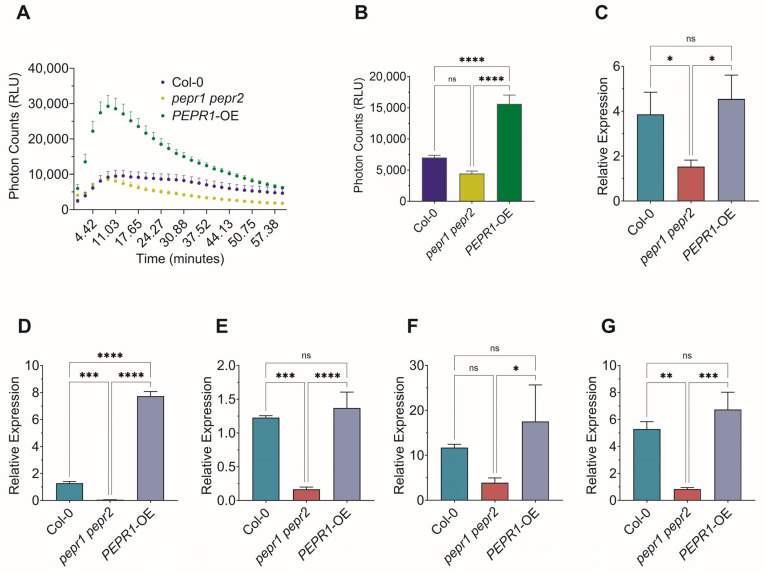
PEPR1 contributes to SsNLP1-induced immune responses. ROS production and transcriptional responses were analyzed in *Arabidopsis* Col-0, the *pepr1 pepr2* double mutant, and *PEPR1*-OE lines following SsNLP1 treatment. (**A**) Kinetics of ROS burst in response to SsNLP1, measured as relative luminescence units (RLU) over time (mean ± SEM, *n* = 8). (**B**) Total ROS production induced by SsNLP1 across genotypes, calculated from the same experiment shown in (**A**) and presented as cumulative RLU over the measurement period. (**C**–**G**) Relative expression levels of defense-related genes following SsNLP1 treatment. Leaf disks were treated with SsNLP1 or water for 1 h, and transcript levels were quantified by qRT-PCR. *ACTIN8* was used as an internal control. Gene expression levels were normalized to the corresponding water-treated controls for each genotype: (**C**) *FRK1*, (**D**) *PDF1*.2, (**E**) *PR1*, (**F**) *WRKY33*, and (**G**) *WRKY53*. Data are presented as mean ± SD (*n* = 3). Statistical significance was determined using one-way ANOVA. Statistical significance is indicated as * *p* < 0.05, ** *p* < 0.01, *** *p* < 0.001, **** *p* < 0.0001, and ns, not significant.

*S. sclerotiorum*-infected leaves subjected to DAB staining showed increased H_2_O_2_ accumulation in SsNLP1-pre-treated *PEPR1*-OE lines compared with Col-0, whereas the *pepr1 pepr2* mutant displayed the weakest staining (Figure 4B, lower panel). Consistently, quantitative ROS burst assays confirmed genotype-dependent differences in ROS production. SsNLP1-induced *PEPR1*-OE lines showed higher relative luminescence units (RLU), while the *pepr1 pepr2* mutant exhibited reduced ROS levels compared with Col-0. Specifically, ROS production in the mutant was lower than that in Col-0 and substantially lower than that in the *PEPR1*-OE lines (Figure 5A,B).

To further examine transcriptional responses associated with SsNLP1 treatment, the expression of selected defense-related genes was analyzed following SsNLP1 treatment (Figure 5C–G). Leaves were treated with SsNLP1 or water for 1 h, and transcript levels of *FRK1*, *PDF1.2*, *PR1*, *WRKY33*, and *WRKY53* were quantified. SsNLP1 treatment resulted in genotype-dependent modulation of gene expression. The PTI marker *FRK1* showed reduced expression in the *pepr1 pepr2* mutant and a modest increase in *PEPR1*-OE lines (Figure 5C). Similarly, *PDF1*.2 expression was suppressed in the mutant but enhanced in *PEPR1*-OE lines (Figure 5D). Expression of *PR1*, *WRKY33*, and *WRKY53* followed comparable trends, with generally reduced expression in *pepr1 pepr2* and moderate increases in *PEPR1*-OE lines (Figure 5E–G).

Collectively, these results indicate that PEPR1 contributes to SsNLP1-induced immune responses such as ROS burst and immune-related gene expression enhancement, suggesting coordination between phytocytokine signaling and PAMP signaling for enhanced plant resistance to *S. sclerotiorum*.

## 3. Discussion

In this study, we provide a comprehensive analysis of the contribution of the *Arabidopsis* LRR-RLK PEPR1 to immune signaling triggered by SsNLP1. SsNLP1 is a *Sclerotinia sclerotiorum* necrosis- and ethylene-inducing peptide 1 (Nep1)-like protein (NLP) that functions as a PAMP to elicit plant immune responses upon recognition by host PRRs [17,20,32]. By combining computational and experimental approaches, including phylogenetic analysis, protein domain characterization, and functional validation using CRISPR-Cas9 mutants and overexpression lines we demonstrate that PEPR1 plays a significant role in amplifying immune responses following SsNLP1 treatment. Our results indicate that PEPR1-mediated signaling contributes substantially to early immune responses, including ROS production and the activation of defense genes, and acts as a positive regulator in the defense against *S. sclerotiorum*. These findings support a model in which SsNLP1 enhances resistance by amplifying host immune signaling, with PEPR1 acting as an important component of this response.

Phylogenetic analysis positioned SsNLP1 within a clade closely associated with *B. cinerea* NLPs, clustering closely with an NLP from *B. cinerea* (Figure 1), which is consistent with the close evolutionary relationship between these two necrotrophic pathogens within the Sclerotiniaceae. This observation is biologically relevant because *B. cinerea* NLPs have been shown to function as immunogenic patterns capable of activating host defense responses [16]. The separation of SsNLP1 and SsNLP2 into distinct clades, with SsNLP1 clustering with a *B. cinerea* NLP (100% bootstrap) and SsNLP2 grouping with *Penicillium chrysogenum* NLP (88% bootstrap), further suggests functional diversification of NLP family members in *S. sclerotiorum*, despite their shared classification as type I NLPs. Although both proteins possess the conserved NPP1 domain and the hallmark GHRHDWE motif (Figure 2A), their relatively low sequence identity (39%) and differences in cysteine composition and net charge indicate possible divergence in stability, membrane interaction, or host recognition properties. Similar diversification among NLP paralogs has been reported in other fungal pathogens and may reflect adaptation to distinct stages of infection or host targets [17,33].

The conserved GHRHDWE motif present in the synthetic SsNLP1 peptide used in this study is particularly important, as residues within this heptapeptide core are required for NLP-induced necrosis [17]. Albert et al. (2015) [18] demonstrated that *Arabidopsis* recognizes conserved nlp20/nlp24 peptides derived from microbial NLPs through the receptor-like protein RLP23 in association with SOBIR1 and BAK1, thereby activating PTI. This recognition mechanism was also shown to function in potato and tomato and upon transgenic expression of *RLP23*. Because the SsNLP1 peptide used here contains this conserved motif, it is likely to function as a PAMP-like elicitor rather than as a necrosis-inducing toxin. Our study did not directly examine RLP23-mediated recognition. However, the observed immune activation is consistent with previous reports. Synthetic nlp20 peptides have been shown to trigger PTI-associated responses, including ROS production, MAPK activation, and defense gene expression. These responses occur without inducing necrosis. This indicates that immune activation can occur independently of the cytotoxic activity of full-length NLPs [14,18].

One of the findings in this study is that SsNLP1 treatment induced the expression of both *PEPR1* and *PEPR2*, with *PEPR1* showing a more pronounced transcriptional increase (Figure 3). PEPR1 and PEPR2 are well-characterized receptors for endogenous AtPep peptides and play important roles in amplifying immune signaling during pathogen attack and tissue damage [24,25]. Their induction by SsNLP1 suggests that NLP-triggered immunity may engage endogenous danger signaling pathways in addition to classical PAMP perception. Similar cross-activation between exogenous pathogen-derived elicitors and endogenous DAMP pathways has been reported for several PRR systems, where initial immune activation promotes PROPEP expression and AtPep release, leading to signal amplification through PEPR signaling [34,35]. The stronger induction of PEPR1 compared with PEPR2 may indicate a more prominent contribution of PEPR1 during early SsNLP1 responses, although functional redundancy between these receptors is well established. This redundancy was an important consideration in our experimental design. Because single *pepr1* or *pepr2* mutants retain substantial immune responsiveness to certain Pep peptides due to partial functional overlap and differential ligand specificities of the two receptors [25], we employed the *pepr1 pepr2* double mutant for functional analysis. PEPR1 shares structural characteristics with canonical immune receptors such as FLS2, including a large extracellular LRR domain [36] and a kinase region, supporting its capacity to function as a central immune-signaling component. The opposing defense profiles observed between the *pepr1 pepr2* and *PEPR1*-overexpression lines, further indicate that PEPR signaling contributes substantially to defense transcriptional reprogramming.

Functionally, *PEPR1* overexpression enhanced SsNLP1-associated resistance to *S. sclerotiorum*, whereas the *pepr1 pepr2* mutant showed increased susceptibility (Figure 4B–D). Both lesion area measurements and fungal biomass quantification consistently demonstrated that SsNLP1 pre-treatment reduced disease severity, with the strongest protection observed in *PEPR1*-OE lines and the weakest response in the double mutant. Because fungal biomass closely mirrored lesion development (Figure 4E), the observed phenotypes are unlikely to reflect only differences in symptom progression and instead support a genuine effect on pathogen colonization.

These findings are consistent with previous reports showing that PEPR-mediated signaling contributes to resistance against biotic stressors, including *P. syringae* pv. *tomato* (*Pst*) [29], the necrotrophic pathogens *Pythium irregulare* [25], *B. cinerea* [28], and herbivores [30], and enhances defense amplification following tissue damage. More recently, the metacaspase-Peps-PEPR immune module was shown to confer resistance to *Fusarium* head blight in wheat [37], demonstrating that PEPR-mediated phytocytokine signaling is functionally conserved in crop species. Importantly, our data do not demonstrate that PEPR1 directly perceives the SsNLP1 peptide. Rather, they indicate that PEPR signaling is required for the full magnitude of SsNLP1-triggered resistance, suggesting that PEPR1 functions downstream of, or in parallel with primary NLP perception pathways.

Early immune responses further supported this interpretation. ROS production was significantly enhanced in *PEPR1*-OE lines and reduced in the *pepr1 pepr2* mutant following SsNLP1 treatment (Figure 5A,B), indicating that PEPR signaling contributes to rapid oxidative burst activation. ROS production is one of the earliest outputs of PRR activation and plays important roles in both antimicrobial defense and signaling amplification [38,39]. PEPR1 has previously been shown to form ligand-induced complexes with BAK1 and activate downstream signaling cascades similar to those of FLS2 and EFR [36]. The enhanced ROS accumulation observed here is therefore consistent with PEPR1 functioning as an amplifier of PAMP-triggered immunity initiated by SsNLP1.

Gene expression analysis revealed coordinated regulation of *FRK1*, *PDF1.2*, *PR1*, *WRKY33*, and *WRKY53* in a PEPR-dependent manner (Figure 5C–G). *FRK1*, a canonical PTI marker [40,41], showed reduced induction in the *pepr1 pepr2* double mutant, supporting impaired early signaling. *PDF1*.2, commonly associated with jasmonic acid/ethylene (JA/ET)-mediated defense against necrotrophs [42], was enhanced in *PEPR1*-OE lines and suppressed in the mutant. This pattern suggests that PEPR signaling contributes to the activation of defense pathways relevant to *S. sclerotiorum* resistance, consistent with previous reports that PEPRs regulate *PDF1.2* expression in response to Pep peptides [43].

WRKY33, a key regulator of resistance against necrotrophic fungi including *B. cinerea* and *Alternaria brassicicola* [44,45], followed a similar pattern, further supporting this interpretation. Because our study did not directly assess hormone accumulation or additional JA/ET pathway regulators such as ERF1 or ORA59, conclusions regarding pathway prioritization should remain cautious. Rather than definitively assigning pathway dominance, our data indicate that PEPR1-associated signaling contributes to both PTI activation and defense outputs compatible with resistance to necrotrophic infection.

Interestingly, *PR1* expression was also affected by PEPR status, despite PR1 being commonly associated with salicylic acid (SA) signaling. This may reflect the known complexity of immune network crosstalk, where PEPR signaling can co-activate both SA- and JA-mediated branches [43]. The strong positive correlations observed among defense genes, particularly between *WRKY33* and *FRK1* (Figure 5C,F), further support coordinated transcriptional regulation rather than strict pathway separation. Such integration may be particularly important during interactions with necrotrophs, where excessive cell death can benefit the pathogen and balanced immune regulation is essential.

Overall, our findings support a model in which the SsNLP1-derived peptide acts as an immunogenic elicitor that activates *Arabidopsis* defense responses and contributes to enhanced resistance against *S. sclerotiorum*. PEPR1-associated signaling strengthens this response through amplification of ROS production and defense gene activation. We propose that PEPR1 functions as an important component of downstream immune reinforcement, potentially linking RLP23-mediated NLP recognition with endogenous PROPEP/AtPep-dependent danger signaling. Future work should clarify how PEPR1-associated signaling integrates with the established RLP23-mediated recognition of NLP-derived peptides. In particular, it remains unclear whether SsNLP1 perception through RLP23 promotes PROPEP induction and subsequent AtPep release, thereby activating PEPR-dependent immune amplification. Biochemical and genetic studies addressing receptor complex formation, ligand specificity, and signaling hierarchy will be necessary to define the precise relationship between RLP23-triggered perception and PEPR-mediated defense amplification during *S. sclerotiorum* infection. Notably, recent work has demonstrated that fungal pathogens can manipulate phytocytokine signaling to promote infection [46], highlighting the central role of phytocytokine pathways in plant–pathogen interactions.

## 4. Materials and Methods

### 4.1. Sequence Retrieval and Database Mining

Protein sequences of SsNLP1 (accession: XP_001596857.1; 246 amino acids) and SsNLP2 (accession: XP_001586883.1; 245 amino acids) from *S. sclerotiorum* strain 1980 (NCBI Taxonomy ID: 665079) were retrieved from the National Center for Biotechnology Information (NCBI) protein database (https://www.ncbi.nlm.nih.gov/protein/ (accessed on 10 April 2026)). Orthologous NLP sequences from diverse fungal taxa were obtained using BLASTp (version 2.17.0+) searches against the NCBI non-redundant (nr) protein database with the SsNLP1 sequence as the query, applying an E-value threshold of 1 × 10^−5^, a minimum query coverage of 70%, and a sequence identity > 25%. The following organisms were included for comparative analysis: *B. cinerea* (Leotiomycetes), *F. oxysporum* (accession AAC97382.1; Sordariomycetes), *V. dahliae* (UniProt Q6QUY0; Sordariomycetes), *Pyricularia oryzae* (accession KAI7917644.1; Sordariomycetes), *Colletotrichum gloeosporioides* (Sordariomycetes), *Cladobotryum mycophilum* (Sordariomycetes), *Aspergillus destruens*, *A. magnivesiculatus*, *A. vitricola* (Eurotiomycetes), *Penicillium chrysogenum* (Eurotiomycetes), *Cladosporium cladosporioides* (Dothideomycetes), *F. fulva* (Dothideomycetes), *Phytophthora parasitica* (UniProt Q9AT28; Oomycota), and *Pythium aphanidermatum* (UniProt Q9SPD4; Oomycota). The initial BLASTp search returned several hundred hits. To select a representative and non-redundant set, we applied the following filtering criteria: (i) only sequences annotated as “necrosis-inducing protein” or “necrosis- and ethylene-inducing peptide” were retained, while unrelated proteins were excluded based on domain verification; (ii) one representative genome per species was used to avoid strain-level redundancy; (iii) exact duplicates and partial sequences (<200 amino acids) were removed; and (iv) sequences exceeding 400 amino acids were excluded as likely fusion proteins, yielding a final set of 26 NLP sequences representing all identified NLP family members from 15 species across two kingdoms (Fungi and Oomycota). All NLP paralogs per species were retained to capture the complete NLP repertoire of each pathogen [24,36].

### 4.2. Phylogenetic Analysis

Multiple sequence alignment and phylogenetic analysis were performed using the Biopython library (v1.84) in Python 3.12 [47]. Pairwise global alignments were computed using the PairwiseAligner module with the following parameters: alignment mode, global; gap-opening penalty, −10; gap-extension penalty, −0.5; and match score, default substitution matrix. An identity-based distance matrix was calculated from pairwise alignments, where the distance between sequences i and j was defined as d(i,j) = 1 − (number of identical residues/alignment length). A neighbor-joining (NJ) phylogenetic tree was constructed from the distance matrix using the DistanceTreeConstructor module in the Bio.Phylo.TreeConstruction package. The tree was rooted at the midpoint using the root_at_midpoint function. Tree visualization was performed using matplotlib (v3.10.8) with custom color-coding by taxonomic group. Branch support was assessed by bootstrap analysis with 1000 replicates using parametric resampling of the distance matrix. Bootstrap values (%) were displayed at internal nodes for clades with support ≥ 50%.

### 4.3. Protein Domain and Motif Analysis

Protein domain architectures of SsNLP1 and SsNLP2 were annotated by integrating information from multiple sources. Signal peptide cleavage sites were predicted using SignalP 6.0 (eukaryotic mode) and verified with Phobius via the InterProScan 5 web server (https://www.ebi.ac.uk/interpro/ (accessed on 13 April 2026)). The conserved GHRHDWE heptapeptide motif, diagnostic of the necrosis-inducing phytotoxin (NPP1) domain (Pfam: PF05630), was identified by scanning each sequence for the exact motif string using custom Python scripts. Cysteine residue positions were mapped across both paralogs to identify conserved disulfide bond-forming pairs characteristic of Type I NLPs. NPP1 domain boundaries (Pfam: PF05630) were annotated using InterProScan 5 with the Pfam database. Domain predictions were cross-validated against the structurally characterized PaNLP from *Pythium aphanidermatum* (PDB: 3GNU) [17,19,48,49].

Amino acid composition analysis was performed by calculating the frequency of each of the 20 standard amino acids as a percentage of total protein length. Physicochemical properties were computed based on amino acid residue properties: molecular weight was calculated as the sum of individual amino acid molecular weights minus (n − 1) × 18 Da for peptide bond formation; net charge was estimated by counting positively charged (Lys, Arg, and His) and negatively charged (Asp and Glu) residues at neutral pH; and hydrophobicity was calculated as the percentage of hydrophobic residues (Ala, Ile, Leu, Met, Phe, Trp, and Val) relative to the total sequence length. Pairwise sequence identity between SsNLP1 and SsNLP2 was computed from global alignments as described above.

### 4.4. Plant Materials and Growth Conditions

All *Arabidopsis* lines used in this study, including wild-type (WT), overexpression (OE), and loss-of-function mutant plants, were in the Columbia-0 (Col-0) background. The *pepr1 pepr2* double mutant was kindly provided by Professor Jianfeng Li, Zhongshan University, China [31].

*Arabidopsis* seeds were surface-sterilized and sown on half-strength Murashige and Skoog (MS) agar medium [50] supplemented with or without 25 μg mL^−1^ hygromycin B. The plates were stratified in darkness at 4 °C for 2 days and then transferred to a growth chamber maintained at 21 °C with 70–80% relative humidity under a 14 h light/10 h dark photoperiod. After 7 days, seedlings were transplanted into pots containing Sunshine soil and grown under the same environmental conditions.

### 4.5. Vector Construction and Plant Transformation

To generate *PEPR1* (AT1G73080) OE lines, the full-length coding sequence (CDS) of *PEPR1* was amplified from *Arabidopsis* Col-0 cDNA and cloned into the binary vector pFGC1008-HA under the control of the Cauliflower Mosaic Virus (CaMV) 35S promoter. The resulting construct was introduced into *Agrobacterium tumefaciens* strain GV3101 and transformed into *Arabidopsis* plants using the floral dip method [51]. Transgenic seedlings were selected on half-strength MS medium containing hygromycin B, and positive transformants were further confirmed by PCR using gene-specific and vector-specific primers.

### 4.6. Peptide

The *S. sclerotiorum* necrosis- and ethylene-inducing peptide 1 (SsNep1)-like peptide 1 (SsNLP1) (sequence: GIMYAWYFPKDQPAAGNVVGGHRHDWE) was chemically synthesized by China Peptides. The synthesized peptide was dissolved in sterile distilled water, and a working concentration of 1 μM was used for all experiments conducted in this study.

### 4.7. Plant Inoculation Assay

Fresh sclerotia of *S. sclerotiorum* strain UF1 [52] were cultured on potato dextrose agar (PDA) at 23 °C. After 2 days of incubation, agar plugs containing actively growing mycelia emerging from germinated sclerotia were transferred to fresh PDA plates and incubated for an additional 36 h. This established a newly expanding hyphal growth zone at the colony margin. Twenty-four hours prior to pathogen inoculation, leaves from each *Arabidopsis* genotype were infiltrated with either 1 μM SsNLP1 or sterile distilled water as a control. This allowed establishment of a stable immune priming response prior to infection. This timing is consistent with previous optimization of SsNLP-mediated resistance responses in *Arabidopsis* [20]. Subsequently, mycelial plugs were excised from the actively growing colony margins (new hyphal front) and placed onto the pretreated leaves for inoculation. Inoculated plants were maintained in a growth chamber under high-humidity conditions at 21 °C. Disease progression was assessed by measuring lesion area using ImageJ software (version 1.54g, https://imagej.net/ij/ (accessed on 20 April 2025)). For each genotype and treatment, a minimum of 10 plants were analyzed to evaluate disease resistance.

### 4.8. Measurement of Reactive Oxygen Species (ROS)

Detection of hydrogen peroxide (H_2_O_2_) accumulation was performed using 3,3′-diaminobenzidine (DAB) staining with minor modifications to a previously described method [53]. *Arabidopsis* leaves inoculated with *S. sclerotiorum* were immersed in DAB solution (1 mg mL^−1^, pH 7.0) for 3 h. Following staining, chlorophyll was removed by incubating the leaves in 95% ethanol with gentle shaking until complete decolorization was achieved. The formation of reddish-brown precipitates was used as an indicator of H_2_O_2_ accumulation, and images were captured under white light.

Quantitative analysis of ROS production was conducted largely as previously described [20]. Leaf disks (3 mm in diameter) were excised from all *Arabidopsis* genotypes and incubated overnight in sterile water in 96-well plates under dark conditions. On the following day, the water was replaced with a reaction solution containing 20 μg mL^−1^ horseradish peroxidase (HRP; Sigma-Aldrich, St. Louis, MO, USA), 11 μM L-012 (FUJIFILM Wako Pure Chemical, Osaka, Japan), and 1 μM SsNLP1 peptide. Luminescence was measured over the indicated time intervals using a Microplate Luminometer (Berthold Technologies, Bad Wildbad, Germany). ROS accumulation was quantified by monitoring changes in relative light units (RLU) over time or by calculating the total integrated RLU values.

### 4.9. Fungal Biomass Determination

Leaves from all *Arabidopsis* genotypes and treatment groups inoculated with *S. sclerotiorum* were collected for genomic DNA extraction. Total DNA was isolated using the cetyltrimethylammonium bromide (CTAB) method, followed by RNase A treatment to eliminate RNA contamination. The purified genomic DNA was used as the template for quantification of fungal biomass by quantitative real-time PCR (qRT-PCR). Reactions were performed using AceQ qPCR SYBR Green Master Mix (Vazyme, Nanjing, China) on a StepOne Real-Time PCR System (Applied Biosystems, Foster City, CA, USA). The relative abundance of *S. sclerotiorum* was determined by amplification of the *SsITS* gene and calculated using the 2^−ΔΔCt^ method, with *AtRuBisCO* used as the internal reference gene. Primer sequences are provided in Supplementary Table S1.

### 4.10. Gene Expression Analysis

Leaves of *Arabidopsis* infiltrated with either SsNLP1 or sterile water were harvested for total RNA extraction. Total RNA was isolated using FreeZol Reagent (Vazyme, Nanjing, China) according to the manufacturer’s protocol. First-strand cDNA synthesis was performed using the HiScript II 1st Strand cDNA Synthesis Kit (+gDNA Wiper), (Vazyme, Nanjing, China) following the supplier’s instructions. The qRT-PCR was carried out using AceQ qPCR SYBR Green Master Mix (Vazyme, Nanjing, China) on either a LightCycler 96 (Roche Molecular Systems, Pleasanton, CA, USA) or a StepOne Real-Time PCR System (Applied Biosystems, Foster City, CA, USA). Relative transcript levels of target genes were normalized against *ACTIN8* and calculated using the 2^−ΔΔCt^ method. Primer sequences used for qRT-PCR analysis are listed in Supplementary Table S1. All primers were validated for specificity by melt curve analysis.

## Figures and Tables

**Table 1:** Distinctive features of the three phylogenetic NLP groups.

Feature	Group I	Group II	Group III
Members	SsNLP1, BcNLP1, FoNLP1, VdNLP1, VdNLP1b, VdNLP2, CcNLP1	SsNLP2, BcNLP2, FoNLP2, CcNLP2, CgNLP1, FfNLP1, PoNLP1, PpNLP1, PaNLP1, AvNLP1-3, AdNLP1-2, AmNLP1, ApNLP1	FoNLP3, VdNLP3, CmNLP1
Taxonomy	Necrotrophic Leotiomycetes and Sordariomycetes	Oomycetes, Aspergillaceae, second paralogs, single NLPs	Third paralogs (Sordariomycetes) and *Cladobotryum mycophilum*
GHRHDWE motif	Present in all (7/7)	Present in 15/16; absent in FfNLP1	Present in 2/3; absent in CmNLP1
Serine content	5.7 ± 0.4%	10.0 ± 1.3%	5.5 ± 0.9%

## Data Availability

The original contributions presented in the study are included in the article/Supplementary Materials. Further inquiries can be directed to the corresponding authors.

## Supplementary Materials

The following supporting information can be downloaded at: https://www.mdpi.com/article/10.3390/ijms27125271/s1.

## Abbreviations

The following abbreviations are used in this manuscript:

AtPep	*Arabidopsis* plant elicit peptide
BAK1	Brassinosteroid insensitive 1-associated kinase 1
BIK1	Botrytis-induced kinase 1
CERK1	Chitin elicitor receptor kinase 1
CTAB	Cetyltrimethylammonium bromide
CWDEs	Cell wall-degrading enzymes
DAMPs	Damage-associated molecular patterns
ETI	Effector-triggered immunity
FLS2	Flagellin-sensing 2
FRK1	Flg22-induced receptor-like kinase 1
HA	Hemagglutinin tag
HRP	Horseradish peroxidase
ITS	Internal transcribed spacer
JA	Jasmonic acid
LRR	Leucine-rich repeat
LRR-RLK	Leucine-rich repeat receptor-like kinase
LYK5	Lysin motif receptor kinase 5
MAMPs	Microbe-associated molecular patterns
MS	Murashige and Skoog medium
NLPs	Necrosis- and ethylene-inducing peptide 1-like proteins
NLRs	Nucleotide-binding leucine-rich repeat receptors
NJ	Neighbor-joining
NPP1	Necrosis-inducing Phytophthora protein 1 domain
OE	Overexpression
PAMPs	Pathogen-associated molecular patterns
PCA	Principal component analysis
PDA	Potato dextrose agar
PDF1.2	Plant defensin 1.2
PEP	Plant elicitor peptide
PEPR1	Pep1 receptor 1
PEPR2	Pep1 receptor 2
PR1	Pathogenesis-related gene 1
PRRs	Pattern recognition receptors
PTI	Pattern-triggered immunity
qPCR	Quantitative polymerase chain reaction
qRT-PCR	Quantitative real-time polymerase chain reaction
RLPs	Receptor-like proteins
RLKs	Receptor-like kinases
RLU	Relative light units
ROS	Reactive oxygen species
SA	Salicylic acid
SOBIR1	Suppressor of BIR1-1
TAIR	The Arabidopsis information resource
TM	Transmembrane domain
WT	Wild-type
WRKY	WRKY transcription factor family
