# Supplementary material for: PEPR1 Mediates SsNLP1-Triggered Immunity Against Sclerotinia sclerotiorum
Source: Int J Mol Sci. 2026 Jun 10;27(12):5271. doi: 10.3390/ijms27125271 (PMC13300671; doi:10.3390/ijms27125271)
Supplement: Supplementary file 1 [file ijms-27-05271-s001.zip › ijms-4351922-supplementary.pdf]

Supplementary Table S1. Primers used in this study

Gene Name	Primer Name	Sequence (5'-3')
ACTIN8	Actin-8 F	CGAGGCTCCTCTTAACCCAAA
	Actin-8 R	GGCACAGTGTGAGACACACCA
FRK1	FRK1-F	AGGAATCGTGGATCAGCGTC
	FRK1-R	TGACTCATCGTTGGCCTCTG
PDF1.2	PDF1.2-F	CTTTCGACGCACCGGCAATG
	PDF1.2-R	AGTTGCATGATCCATGTTTGGCT
PEPR1	PEPR1-F	CGGCTACATTGCACCAGAAAAC
	PEPR1-R	CCACCGCTCTCTTCCTCGTA
PEPR2	PEPR2-F	TGCGGATAGCTCGAAATGCT
	PEPR2-R	TGTGGTTGGAATCTCACCCG
PR1	PR1-F	CGGGGAAAACTTAGCCTGGG
	PR1-F	ACGTGTTCGCAGCGTAGTTG
Rubisco	Rubisco-F	GCAAGTGTTGGGTTCAAAGCTGGTG
	Rubisco-R	CCAGGTTGAGGAGTTACTCGGAATGCTG
ITS	Ss-ITS-F	GGATCTCTTGGTTCTGGCAT
	Ss-ITS-R	GCAATGTGCGTTCAAAGATT
WRKY33	WRKY33-F	CCATCGGTTGTCCAGTGAGG
	WRKY33-R	TTTGTGGCGTAACCGCTACC
WRKY53	WRKY53-F	CAGAGATCAGACGGGGATGC
	WRKY53-R	TGACTCTGGTGTCTTGTCGC
